# Exploring MRI Characteristics of Brain Diffuse Midline Gliomas With the H3 K27M Mutation Using Radiomics

**DOI:** 10.3389/fonc.2021.646267

**Published:** 2021-05-24

**Authors:** Qian Li, Fei Dong, Biao Jiang, Minming Zhang

**Affiliations:** Department of Radiology, The Second Affiliated Hospital, Zhejiang University School of Medicine, Hangzhou, China

**Keywords:** diffuse midline glioma, H3 K27M mutant, radiomics, magnetic resonance imaging, principal component analysis

## Abstract

**Objectives:**

To explore the magnetic resonance imaging (MRI) characteristics of brain diffuse midline gliomas with the H3 K27M mutation (DMG-M) using radiomics.

**Materials and Methods:**

Thirty patients with diffuse midline gliomas, including 16 with the H3 K27M mutant and 14 with wild type tumors, were retrospectively included in this study. A total of 272 radiomic features were initially extracted from MR images of each tumor. Principal component analysis, univariate analysis, and three other feature selection methods, including variance thresholding, recursive feature elimination, and the elastic net, were used to analyze the radiomic features. Based on the results, related visually accessible features of the tumors were further evaluated.

**Results:**

Patients with DMG-M were younger than those with diffuse midline gliomas with H3 K27M wild (DMG-W) (median, 25.5 and 48 years old, respectively; p=0.005). Principal component analysis showed that there were obvious overlaps in the first two principal components for both DMG-M and DMG-W tumors. The feature selection results showed that few features from T2-weighted images (T2WI) were useful for differentiating DMG-M and DMG-W tumors. Thereafter, four visually accessible features related to T2WI were further extracted and analyzed. Among these features, only cystic formation showed a significant difference between the two types of tumors (OR=7.800, 95% CI 1.476–41.214, p=0.024).

**Conclusions:**

DMGs with and without the H3 K27M mutation shared similar MRI characteristics. T2W sequences may be valuable, and cystic formation a useful MRI biomarker, for diagnosing brain DMG-M.

## Introduction

Diffuse midline gliomas with the H3 K27M mutation (diffuse midline glioma, H3 K27M-mutant) (DMG-M) is a newly defined entity in the 2016 World Health Organization (WHO) classification of central nervous system tumors (1). It describes a group of tumors with mutations in either H3F3A or HIST1H3B/C (2, 3). The term DMG-M is suggested to be only used for tumors that are diffuse, midline gliomas with an H3 K27M mutation, and not for other tumors with the H3 K27M-mutant. A previous study showed that patients with DMG-M had a worse prognosis (with a 2-year survival rate of less than 10%) than those with a diffuse midline glioma, H3 K27M wild (DMG-W) regardless of age, tumor location, or histopathological grading (3, 4). Another advantage for identifying H3 K27M status is that it may be a potentially novel target for immunotherapy for diffuse midline glioma (DMG) (2, 5–7). However, because diffuse midline gliomas are usually located at deep anatomic sites, surgical resection or biopsy is challenging because of the substantial perioperative risks and postoperative morbidities (1). Therefore, developing a non-invasive method for diagnosing DMG-M would be highly valuable (1).

Magnetic resonance imaging (MRI) is an essential technology for the evaluation of brain tumors. Traditionally, visually accessible MRI features are often used for brain tumor evaluation. Identifying important features is often done using experience, such as reading a group of images of patients and summarizing the findings. In addition, a feature set, such as the Visually Accessible Rembrandt Images (VASARI) feature set, is sometimes applied to explore the imaging characteristics of a disease by systematically testing individual features. A previous study found that there were no differences between DMG-M and DMG-W tumors regarding visually accessible MRI features (8). However, it is unclear whether these features are sufficient to represent the characteristics of this disease.

Radiomics is a novel method for high-throughput extraction of quantitative features from a specified region of interest from images (1, 9). These features include many groups, such as shape-based, first-order and texture features. For shape-based features, radiomics not only extracts size and volume data, but also provides additional information such as degree of sphericity and surface area. First-order features provide intensity distribution information such as asymmetry (skewness) and flattening (kurtosis), and texture features provide a more in-depth analysis of the relationships between voxels (10). Analyzing these features might provide a more comprehensive method for exploring a lesion. In recent years, radiomics has been widely used for the classification of phenotypes and genotypes, as well as to predict disease progression (11).

We believe that the analysis of radiomic features may be highly useful for exploring the imaging characteristics of DMG-M and may further guide us in finding useful visually accessible features of this type of tumor. Thus, the aim of this study was to explore the MRI characteristics of brain DMG-M using radiomics.

## Materials and Methods

### Patients

This study was approved by the Local Ethics Committee of our hospital, and the requirement for patient informed consent was waived due to the retrospective character of the study. Thirty patients with diffuse midline gliomas with pathologically confirmed H3 K27M status by immunohistochemistry from January 2017 to October 2020 were retrospectively collected in this study. For all patients, the preoperative Karnofsky Performance Score (KPS) was evaluated when they were admitted to the hospital. The overall survival (OS) time was measured from the time of diagnosis to death or to the last follow-up (censored) (12).

### MRI Protocol

MRI was performed with 1.5T Sonata, Aera, Avanto scanner (Siemens Medical Solutions), 3.0T Discovery 750, Signa HDxt scanner (GE Healthcare) and uMR 790 scanner (United Imaging Healthcare). Axial T1-weighted images (T1WI), T2-weighted images (T2WI) and contrast enhanced T1-weighted images (CeT1WI) were acquired with the following parameters: T1WI and CeT1WI (repetition time (TR), 400–2096.3 ms, and echo time (TE), 6.9–26.5 ms), T2WI (TR, 2,800–4,730 ms, and TE, 83–116 ms), and all the images were acquired with a section thickness of 6 mm. Pre-contrast Gadodiamide (Omniscan, GE Healthcare) was injected through a peripheral venous catheter at a dose that was standardized based on patient body weight (0.2 ml/kg body weight, up to a maximum of 20 ml).

### Image Preprocessing and Segmentation

To reduce discrepancies caused by different MR image acquisition conditions, a series of image preprocessing steps were performed. First, the T1W and CeT1W images were co-registered to the corresponding T2W images using a rigid transformation (13). Denoising was then performed for all images. To compensate for intensity non-uniformities due to variations in the magnetic field, an N4 bias field correction was performed (14, 15). The hybrid white-stripe method was then used for signal intensity normalization (16). Finally, the images were resampled to 3 mm × 3 mm × 3 mm voxels using a sitkBSpline interpolator. Preprocessing was performed using the ITK-SNAP software (http://www.itksnap.org), Cancer Imaging Phenomics Toolkit (17, 18), and Pyradiomics (http://www.radiomics.io/pyradiomics.html).

**Figure 1:**
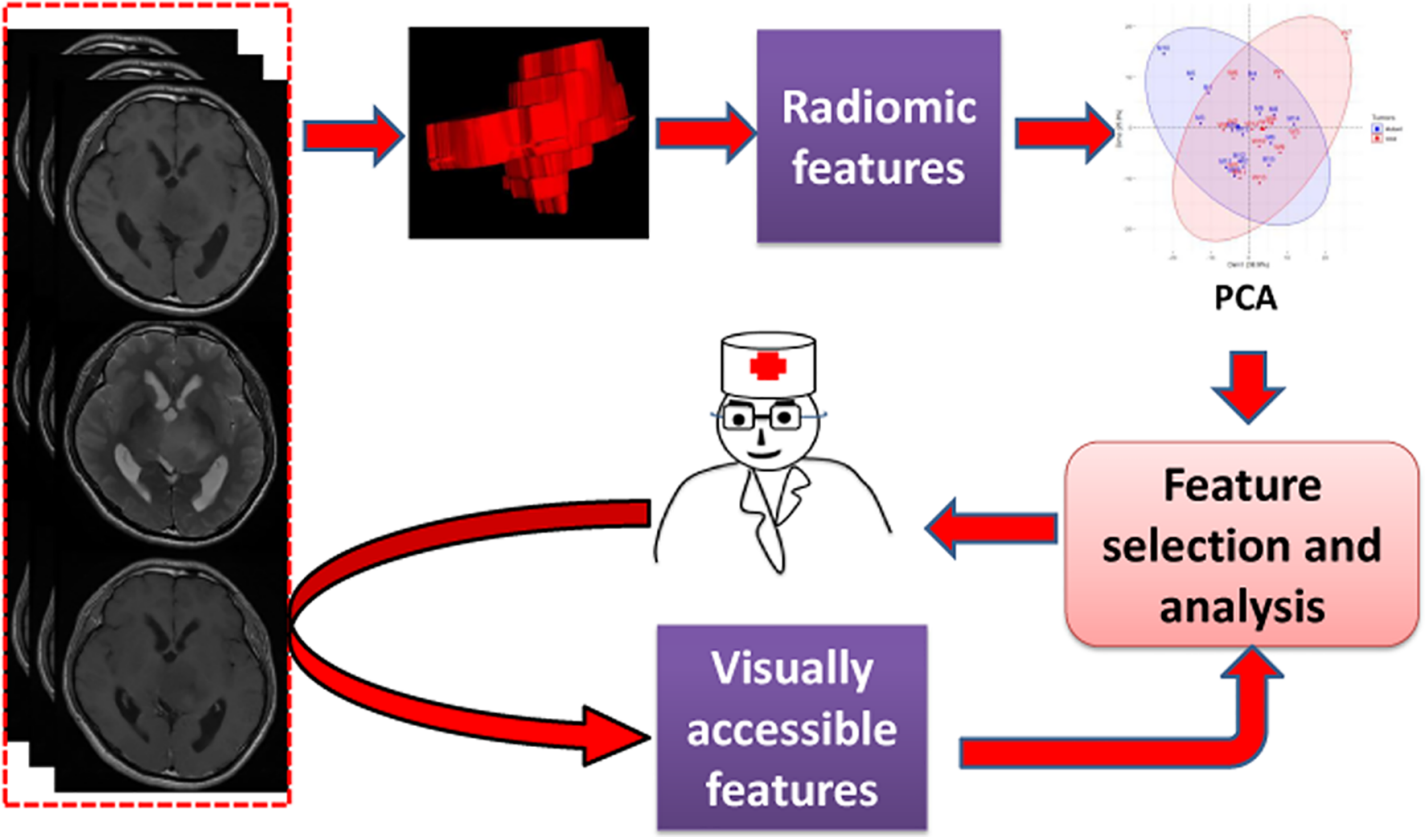
The workflow of this study.

Manual segmentation of the tumor for all cases was performed by a radiologist (F.D., with 10 years of experience) on T2WI, T1WI, and CeT1WI in a sequential manner one slice at a time. The segmentation for all cases was then repeated by another radiologist (Q.L., with 7 years of experience). To identify tumor boundary, the tumors were defined as regions with high signal intensity on T2WI but with less than that of cerebrospinal fluid, and with corresponding T1WI hypointensity (**Figure 1**).

### Feature Extraction

Radiomic features were extracted using Pyradiomics from axial T1WI, T2WI, and CeT1WI. Seven groups of features were extracted, including shape features (n = 14), first-order features (n=18), gray level co-occurrence matrix (GLCM) features (n=22), gray-level run length matrix (GLRLM) features (n = 16), gray-level size zone matrix (GLSZM) features (n=16), gray level dependence matrix (GLDM) features (n = 14). The extracted features were consistent with the Imaging Biomarker Standardization Initiative (IBSI) (19).

The visually accessible features were selectively extracted based on the results of the analysis of radiomic features. We selected visually accessible features from a comprehensive feature set, known as the Visually Accessible Rembrandt Images (VASARI) (https://wiki.cancerimagingarchive.net/display/Public/VASARI+Research+Project), which was specially designed to describe the MR features of human gliomas.

### Reproducibility Evaluation of Features

For radiomic features, intraclass correlation coefficient (ICC) values were calculated to evaluate reproducibility. Features with an ICC value ≥0.90 (13, 20) were retained in this study. For visually accessible features (if they were used), inter-reader agreement was evaluated by calculating the κ values; κ values > 0.81, 0.61 to 0.80, and < 0.60 reflected excellent, good, and poor agreement, respectively (21).

### Exploring MRI Characteristics

To explore the overall characteristics of the tumors, principal component analysis (PCA) of radiomic features was performed. PCA is a type of unsupervised exploratory method, and its main purpose is to transform correlated metric variables into principal components (PCs) that still contain most of the information from the original variables. It is an efficient method for preliminary analysis to determine the number of factors (22). Because we wanted to determine whether differences in imaging characteristics of overall, shape and in each sequence existed between the two kinds of tumors, PCA was implemented in five datasets, including the retained radiomic features (all features) and shape features as well as first-order and texture features in T1WI (T1WI features), T2WI (T2WI features), and CeT1WI (CeT1WI features). The first two or more components were selected, ensuring that at least 60% of the total variance was explained.

Univariate analysis (Student’s t-test or Mann–Whitney U test, as appropriate) was used to explore features with significant differences between DMG-M and DMG-W tumors. A correlation analysis between the significant features was also performed. The number of pairwise correlations of features with |rho| ≥0.90 was regarded as highly correlated (23).

To further explore the MRI features, three feature selection methods were used to select the radiomic features with significant differences between DMG-M and DMG-W tumors. Feature selection methods included variance thresholding, recursive feature elimination, and the elastic net. These methods employ the filter, wrapper, and embedded feature selection methods, respectively. The variance thresholding method first calculates the variance of each feature and then removes features with a variance lower than the threshold. The recursive feature elimination method ranks all of the features from high to low *via* a model, and removes redundant unrelated features (24). The elastic net method is regarded as a combination of the ridge and LASSO (least absolute shrinkage and selection operator) regression methods. It performs better than LASSO in selecting features with multicollinearity (25, 26). In this study, a variance threshold of 0.8 was used for the variance threshold method (27), a support vector machine-based algorithm was used for the recursive feature elimination method (24), and the parameters lambda and alpha (0 to 1, steps of 0.1) were selected using 10-fold cross-validation *via* the minimum-plus-one standard error criterion for the elastic net method (28).

Visually accessible features (if they were used) were evaluated by Fisher’s exact test or Fisher-Freeman-Halton test to explore the differences between DMG-M and DMG-W tumors.

### Statistical Analyses

The demographic characteristics between patients with DGM-M and those with DGM-W tumors were compared using Pearson chi-square test, Fisher’s exact test, Student’s t-test, or the Mann–Whitney U test, as appropriate. The Kaplan-Meier method was used to estimate OS, and the log-rank method was used to compare survival differences between DGM-M and DGM-W patients. Survival analysis was conducted using Cox regression for variate analysis. All statistical tests were two-sided with statistical significance set at p < 0.05. Fisher’s exact test or Fisher-Freeman-Halton test was performed using SPSS19.0. Feature selection with variance thresholding method was implemented by Python (Python 3.6.3, https://www.python.org/). The other data analysis was performed with R software (R 3.6.3, http://www.Rproject.org). The main packages used were: “irr”, “FactoMineR”, “factoextra”, “caret”, “e1071”, “glmnet”, “doParallel”, “dplyr”, “corrplot”, “psy”, “survival”, “survminer”, “qqman”.

## Results

### Clinical Characteristics and Follow-up

A total of 30 patients (18 men and 12 women) were included in the study. Their ages ranged from 8 to 75 years. DMG-M tumors were found in 16 patients (10 men and 6 women), and DMG-W tumors were found in 14 patients (8 men and 6 women). The median time from symptom onset to MR scanning was one month (range, 0.17–36 months). Among the 30 patients, two died due to operative complications, and one was lost to follow-up. The other 27 patients had a median follow-up time of 7 months (range, 1–44 months) and were included in the survival analysis. Demographic and clinical data are presented in **Table 1**.

**Table 1:** Results of the analysis of basic demographic and clinical data.

			DMG-M	DMG-W	p value
Gender	Male	10		6	0.702^*^
	Female	6		6	
Age, median (range)			25.5 (8–58)	48 (18–75)	0.005^#^
Location	Thalamus		10	7	0.713^*^
	Brain stem		6	7
KPS	≤80		7	9	0.722^*^
	>80		5	9
OS, median (months)			22	20	0.400^&^

DMG-M, diffuse midline gliomas, H3 K27M Mutation; DMG-W, diffuse midline gliomas, H3 K27M wild type. Fisher’s exact test (^*^); Mann–Whitney U test (^#^); log-rank test (^&^).

### Extracted Features

A total of 272 radiomic features were initially extracted, including 14 shape features and 86 first-order and texture features for each MR sequence (**Supplemental Data 1**). Thirteen shape features and 196 first-order and texture features (55 from T1WI, 72 from T2WI, and 69 from CeT1WI) showing ICC values ≥0.90 were retained (n = 209).

### Exploring Imaging Characteristics

**Figure 2:**
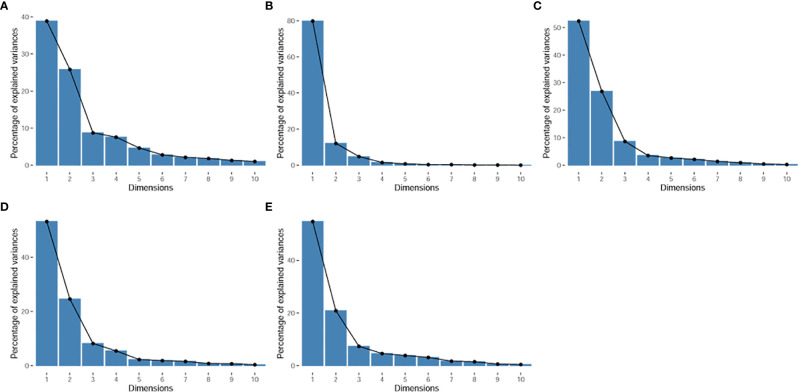
Scree plot. It displays the percentage of explained variances against the number of principal components. **(A)**, for all radiomic features. **(B)**, for shape features. **(C)**, for T1WI features. **(D)**, for T2WI features. **(E)**, for CeT1WI features.

**Figure 3:**
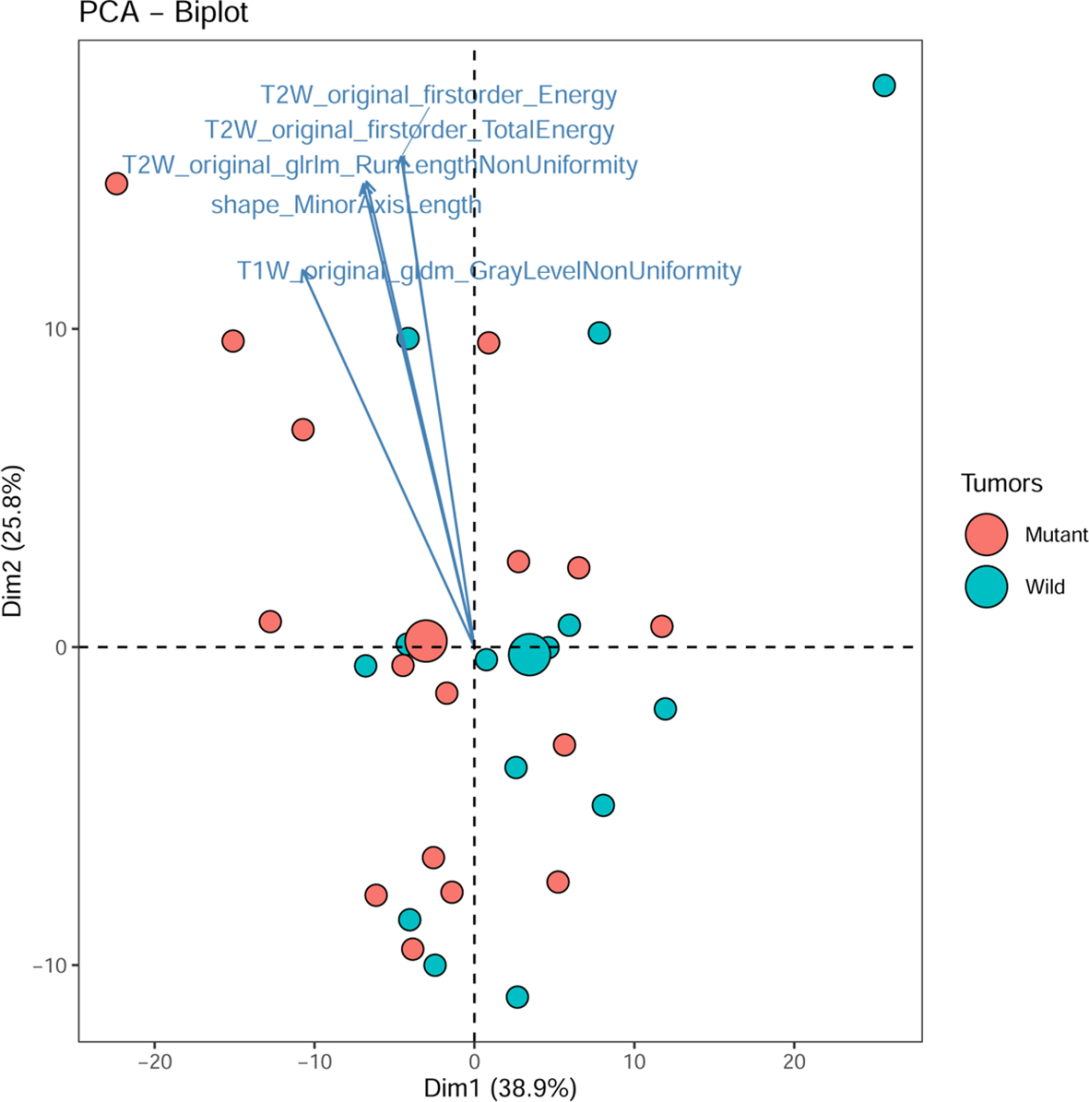
PCA biplot. It shows the contribution of top 5 radiomic features and all individuals to the first two principal components. Mutant, diffuse midline gliomas with the H3 K27M Mutation; Wild, diffuse midline gliomas with the H3 K27M wild.

**Figure 4:**
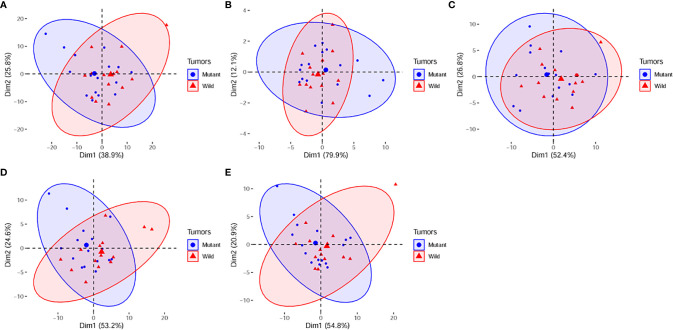
PCA score plots of **(A)**, all radiomic features; **(B)**, shape features. **(C)**, T1WI features. **(D)**, T2WI features. **(E)**, CeT1WI features. PC1, first principal component; PC2, second principal component; Mutant, diffuse midline gliomas with the H3 K27M Mutation; Wild, diffuse midline gliomas with the H3 K27M wild. PCA, principal component analysis; PC, principal component.

The principal component analysis showed that for all features, shape, T1WI, T2WI, and CeT1WI features, the first principal component explained 38.9% to 79.9% of the total variance. The first two principal components explained 64.7% to 92.0% of the total variance, which explained most of the information of the features. Among the top 5 features that contribution to the first two principal components, three of them derived from T2WI. In addition, two cases (cases M10 and W7) were distinct from the others in the principal component, and both tumors were located in the thalamus (**Figures 2**–**4**).

**Figure 5:**
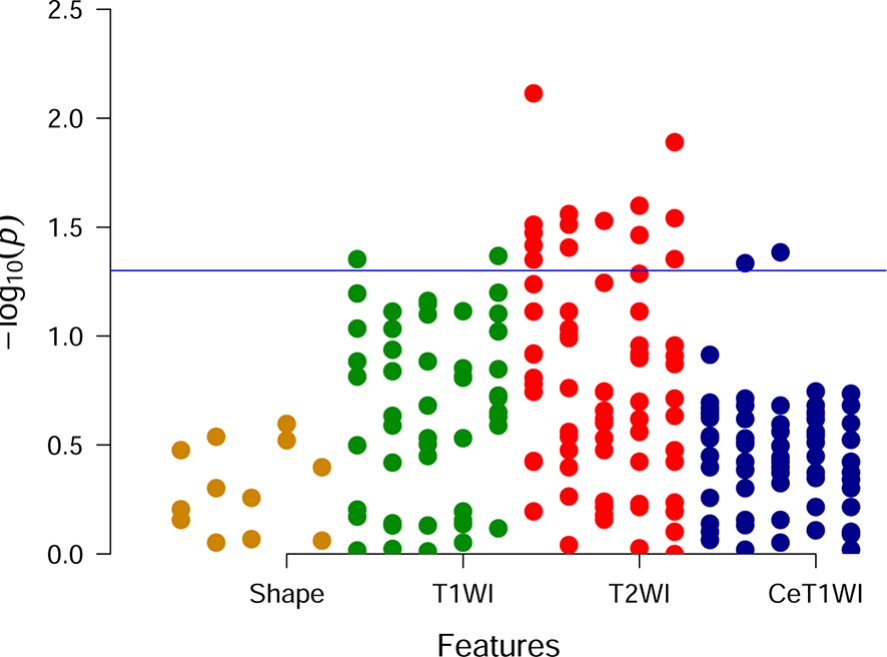
Manhattan plot. Each point is corresponding to a feature and the blue line represents the level of p value equal to 0.05.

**Figure 6:**
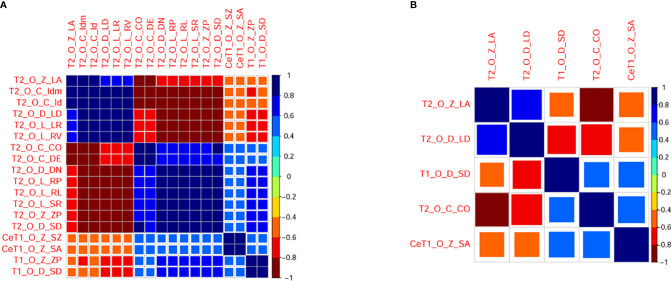
Correlogram illustrating correlations between radiomic features before **(A)** and after **(B)** eliminating redundancy. The larger the square and the darker the color is, the higher the correlation between two features. T1, T1 weighted imaging; T2, T2 weighted imaging, CeT1, contrast enhanced T1 weighted imaging, O, original; Z, GLSZM; D, GLDM; C, GLCM; L, GLRLM; ZP, ZonePercentage; SD, Small Dependence Emphasis; CO, Contrast; DE, Difference Entropy; LR, Long Run Emphasis; RL, Run Length Non Uniformity Normalized; RP, Run Percentage; RV, Run Variance; SR, ShortRunEmphasis; LA, Large Area Low Gray Level Emphasis; DN, Dependence Non Uniformity Normalized; LD, Large Dependence Emphasis; SZ, Size Zone Non Uniformity Normalized; SA, Small Area Emphasis.

Univariate analysis showed that there were 18 features with significant differences between DGM-M and DGM-W tumors (**Figure 5**). Only five features left after discarding the highly correlated features (**Figure 6**), and 60% (three of five) of these features were texture features from T2WI.

**Figure 7:**
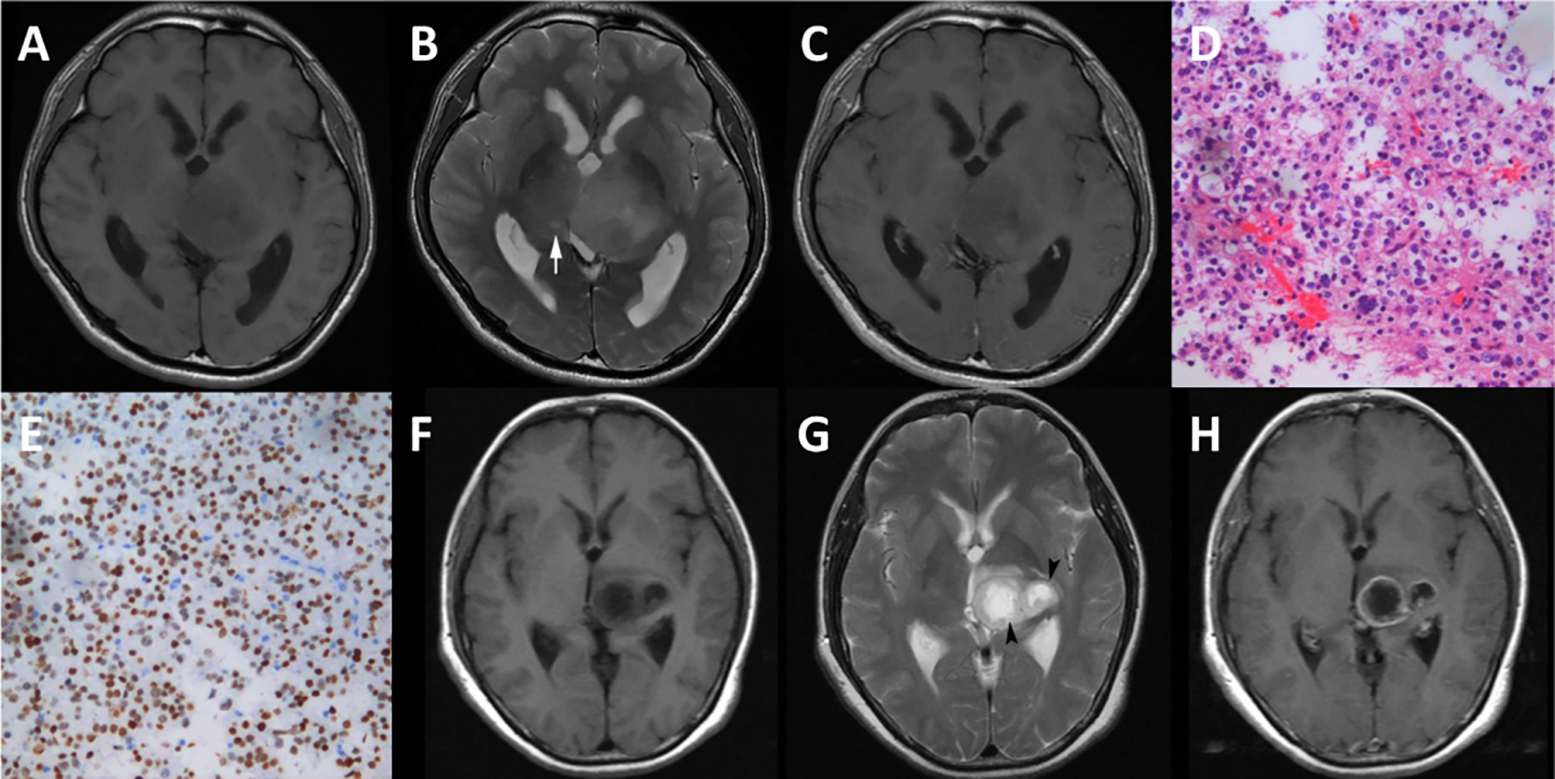
Cases of diffuse midline gliomas. **(A–E)**, a case of diffuse midline gliomas with the H3 K27M mutation. **(F–H)**, a case of diffuse midline gliomas with the H3 K27M wild. **(A, F)**, T1 weighted imaging. **(B, G)**, T2 weighted imaging. **(C, H)**, contrast enhanced T1 weighted imaging. **(D)**, hematoxylin-eosin staining (×20). **(E)**, immunohistological staining (×20) shows H3 K27M positive. cyst formation (white arrow), necrosis (black arrow head).

Although the features selected by the three methods were not identical, all of them were texture features from T2WI (**Table 2**). Therefore, visually accessible features related to T2WI in the VASARI feature set were selected and further analyzed (**Figure 7**).

**Table 2:** Features selection results.

Methods	Features
Variance threshold	T2W_original_glcm_Contrast
	T2W_original_gldm_LargeDependenceEmphasis
Recursive feature elimination	T2W_original_glcm_Contrast
	T2W_original_glszm_LargeAreaLowGrayLevelEmphasis
	T2W_original_glcm_DifferenceEntropy
Elastic net	T2W_original_gldm_SmallDependenceEmphasis

The extracted visually accessible features included cystic formation, necrosis, hemorrhage, and the T1/T2 ratio. A cystic formation was identified as a region that was well defined and usually rounded, showing a low signal on T1WI and high signal on T2WI (higher than the solid part of the tumor and close to cerebrospinal fluid leakage signal intensity), with very thin, regular, and smooth walls. Necrosis was identified in regions within the tumor that had an irregular border, showing a low signal on T1WI, high signal on T2WI. Hemorrhages were defined as foci with a low signal on T2WI or a high signal on T1WI. The T1/T2 ratio was defined as the abnormality size ratio on pre-contrast T1WI and T2WI. The rules for evaluating the features were as follows: (a) cyst formation, 1 = no, 2 = yes; (b) necrosis, 1 = none (0%), 2 = <5%, 3 = 6–33%, 4 = 34–67%, 5 = 68–95% 6 = >95%, 7 = all (100%), 8 = indeterminate; (c) hemorrhage, 1 = no, 2 = yes; (d) T1/T2 ratio, 1 = T1 and T2 approximately the same (T1≈T2), 2 = T1 less than T2 (T1<T2), 3 = T1 far less than T2 (T1<<T2). (https://wiki.cancerimagingarchive.net/display/Public/VASARI+Research+Project).

The inter-reader agreement on visual accessible feature evaluation by the two radiologists was excellent [κ range, (0.84, 0.90)].

Among the visually accessible features, cyst formation showed a significant difference between DGM-M and DGM-W tumors (OR = 7.800, 95% CI 1.476-41.214; p=0.024). There were no significant differences between DGM-M and DGM-W tumors for necrosis (p = 0.191, data in groups 5 and 6 were merged for the limited cases), hemorrhage (p = 0.657), and the T1/T2 ratio (p = 0.689).

### Correlation Between MR Features and Clinical Data

There were no significant differences in symptoms from onset to MR scanning between the two groups with and without cyst formation (p=0.358).

Cox analysis showed that DGM-M type tumor (HR=1.787, p=0.422), older age (HR=1.015, p = 0.540), cystic formation (HR=1.292, p=0.740), larger value of T2W_original_glszm_LargeAreaLowGrayLevelEmphasis (HR=58.848, p=0.277), T2W_original_gldm_LargeDependenceEmphasis (HR=1.181, p=0.105) tended to have a poor OS, while males (HR=0.342, p= 0.168), larger value of T1W_original_gldm_SmallDependenceEmphasis (HR=0.141, 0.685), T2W_original_glcm_Contrast (HR=0.980, p= 0.294) and CeT1W_original_glszm_SmallAreaEmphasis (HR=0.256, p= 0.840) tended to have a good outcome. However, none of these variables were significantly associated with OS.

## Discussion

Identifying DMG-M is critical for treatment decision making, prognosis evaluation. In this study, we explored the MRI characteristics of DMG-Ms using radiomics. Our results showed that although it shared similar characteristics with DMG-W tumors, cyst formation might be a useful MRI characteristic of DMG-M.

Using radiomic analysis, we found that there was an obvious overlap of main principal components in all radiomic features, shape features, and T1WI, T2WI, and CeT1WI features for DMG-M and DMG-W tumors. This indicated that the imaging characteristics of the two types of tumors were similar, and that it may be challenging to differentiate between them.

A previous study used 13 visually accessible features such as size, contrast enhancement pattern, edema, and infiltrative patterns, and found that there was no significant difference in imaging characteristics between DMG-M and DMG-W tumors (8). In fact, even using functional MRI techniques, the authors found no differences in ADC histogram parameters between DGM-M and DGM-W tumors (29). These findings support our PCA results. Therefore, the radiomics-based PCA method may be highly beneficial for initially exploring the overview of imaging characteristics of diseases.

Interestingly, some thalamic DMG tumors showed distinct principal components, indicating that DMG tumors in different locations may have different characteristics, and thus further investigations on DMG tumors according to specific locations may be valuable.

The feature selection results in our study showed that only a few texture features from T2WI were useful for differentiating DMG-M and DMG-W tumors. Similarly, a previous study showed that a gradient boosting classifier, which was built using radiomic features from T2W fluid-attenuated inversion recovery images (FLAIR) was highly efficient in predicting DMG-M (2). However, the specific features selected in our study were different from those of the previous study. Algorithmic differences for feature selection in the two studies may have contributed to these differences. Another reason for differences in feature selection may be attributed to differences between T2WI and FLAIR. Because the range of gliomas may be mismatched on T2WI and FLAIR images, especially for high-grade gliomas (30), the radiomic features derived from the two sequences may be different. It has been reported that DMG-M is a distinct subtype of isocitrate dehydrogenase (IDH) wild-type glioma (31), and that the co-deletion of IDH and 1p/19q are related to T2-FLAIR mismatch (30, 32, 33). Accordingly, we speculate that stratification analysis according to molecular status (such as the presence of an IDH mutant) or T2-FLAIR mismatch may be useful for distinguishing DMG-M and DMG-W tumors, and this therefore warrants further investigation.

We found that among the four visually accessible features, only cyst formation presented significant differences between DMG-M and DMG-W tumors. This was not the first time for cyst formation to be scrutinized. In a previous study, cyst formation was the only feature selected among 13 radiomic and 11 visually accessible features for the identification of high-risk atypical meningiomas (34). Another previous study (8) showed that although there was no significant difference in the cystic component or necrosis between DMG-M and DMG-W tumors, there was a higher ratio of cystic components or necrosis of DMG-M (62.5%) compared to DMG-M (33.3%). Cyst formation is a common feature of gliomas, which may be caused by leakage or secretion of fluid in certain low-grade gliomas (35). Although the mechanism of cyst formation is unclear for diffuse midline gliomas, cyst formation may lead to heterogeneity of the tumor (30). However, our study showed that there was no significant difference in symptom time between the two groups of patients with and without cyst formation, yet cystic formation was not significantly associated with overall survival. This suggests that cyst formation may act as a diagnostic biomarker that is not related to disease course.

This study explored the MR imaging characteristics of DMG-M and found a visually accessible feature that might be useful for identifying this type of tumor. The analysis of a large number of radiomic features using the PCA method and multiple feature selection method provided an overview of the characteristics of the tumor, and may guide us in the selection of special visually accessible features. It may narrow the range of visually accessible features and improved the efficiency of feature selection.

Our study has some limitations. First, the number of subjects included in this study was small, and the ratio of diffuse midline gliomas with the H3 K27M mutation type and those that were wild type might not be representative of the general population. Second, the MR scanning parameters were not the same. Although we performed several preprocessing steps for the images, there might still exist some potential effects on radiomic features. Third, only the original radiomic features and four visually accessible features were used in this study, and more features of the tumor could be explored in the future. Fourth, the follow-up time was short, and our study showed that there was no significant difference in OS between DMG-M and DMG-W tumors. Although previous work has also reported similar results (31), further validation of our results with a large patient cohort is needed.

In conclusion, by using radiomics, our study showed that DMG-M and DMG-W tumors share similar characteristics; however, T2WI and cyst formation may provide useful MR sequences and imaging biomarkers, respectively, for identifying DMG-M tumors.

## Data Availability Statement

The raw data supporting the conclusions of this article will be made available by the authors, without undue reservation.

## Ethics Statement

The studies involving human participants were reviewed and approved by the ethics committee of the Second Affiliated Hospital of Zhejiang University. Written informed consent from the participants’ legal guardian/next of kin was not required to participate in this study in accordance with the national legislation and the institutional requirements.

## Author Contributions

Concept and design: BJ and MZ. Data acquisition: QL and FD. Data analysis and interpretation: QL and FD. Manuscript drafting: FD. Manuscript editing and revising: QL and MZ. Statistical analysis: FD. Administrative and technical support: BJ and MZ. Supervision: MZ. All authors contributed to the article and approved the submitted version.

## Conflict of Interest

The authors declare that the research was conducted in the absence of any commercial or financial relationships that could be construed as a potential conflict of interest.
