# Supplementary material for: Exploring MRI Characteristics of Brain Diffuse Midline Gliomas With the H3 K27M Mutation Using Radiomics
Source: Front Oncol. 2021 May 24;11:646267. doi: 10.3389/fonc.2021.646267 (PMC8182051; doi:10.3389/fonc.2021.646267)
Supplement: Supplementary file 1 [file Table_1.docx]

Supplemental data 1. Features extracted for each imaging sequence

| Type | Features | Number |
| --- | --- | --- |
| Shape | original_shape_Elongation,  original_shape_Flatness,  original_shape_LeastAxisLength, original_shape_MajorAxisLength, original_shape_Maximum2DDiameterColumn, original_shape_Maximum2DDiameterRow, original_shape_Maximum2DDiameterSlice, original_shape_Maximum3DDiameter, original_shape_MeshVolume,  original_shape_MinorAxisLength,  original_shape_Sphericity,  original_shape_SurfaceArea,  original_shape_SurfaceVolumeRatio, original_shape_VoxelVolume | 14 |
| First-order | original_firstorder_10Percentile,  original_firstorder_90Percentile,  original_firstorder_Energy,  original_firstorder_Entropy,  original_firstorder_InterquartileRange,  original_firstorder_Kurtosis,  original_firstorder_Maximum,  original_firstorder_MeanAbsoluteDeviation,  original_firstorder_Mean,  original_firstorder_Median,  original_firstorder_Minimum,  original_firstorder_Range,  original_firstorder_RobustMeanAbsoluteDeviation,  original_firstorder_RootMeanSquared,  original_firstorder_Skewness,  original_firstorder_TotalEnergy,  original_firstorder_Uniformity,  original_firstorder_Variance | 18 |
| GLCM | original_glcm_Autocorrelation,  original_glcm_JointAverage,  original_glcm_ClusterProminence,  original_glcm_ClusterShade,  original_glcm_ClusterTendency,  original_glcm_Contrast,  original_glcm_Correlation,  original_glcm_DifferenceAverage,  original_glcm_DifferenceEntropy,  original_glcm_DifferenceVariance,  original_glcm_JointEnergy,  original_glcm_JointEntropy,  original_glcm_Imc1,  original_glcm_Imc2,  original_glcm_Idm,  original_glcm_Idmn,  original_glcm_Id,  original_glcm_Idn,  original_glcm_InverseVariance,  original_glcm_MaximumProbability,  original_glcm_SumEntropy,  original_glcm_SumSquares | 22 |
| GLRLM | original_glrlm_GrayLevelNonUniformity,  original_glrlm_GrayLevelNonUniformityNormalized,  original_glrlm_GrayLevelVariance,  original_glrlm_HighGrayLevelRunEmphasis,  original_glrlm_LongRunEmphasis,  original_glrlm_LongRunHighGrayLevelEmphasis,  original_glrlm_LongRunLowGrayLevelEmphasis,  original_glrlm_LowGrayLevelRunEmphasis,  original_glrlm_RunEntropy,  original_glrlm_RunLengthNonUniformity,  original_glrlm_RunLengthNonUniformityNormalized,  original_glrlm_RunPercentage,  original_glrlm_RunVariance,  original_glrlm_ShortRunEmphasis,  original_glrlm_ShortRunHighGrayLevelEmphasis,  original_glrlm_ShortRunLowGrayLevelEmphasis | 16 |
| GLSZM | original_glszm_GrayLevelNonUniformity,  original_glszm_GrayLevelNonUniformityNormalized,  original_glszm_GrayLevelVariance,  original_glszm_HighGrayLevelZoneEmphasis,  original_glszm_LargeAreaEmphasis,  original_glszm_LargeAreaHighGrayLevelEmphasis,  original_glszm_LargeAreaLowGrayLevelEmphasis,  original_glszm_LowGrayLevelZoneEmphasis,  original_glszm_SizeZoneNonUniformity,  original_glszm_SizeZoneNonUniformityNormalized,  original_glszm_SmallAreaEmphasis,  original_glszm_SmallAreaHighGrayLevelEmphasis,  original_glszm_SmallAreaLowGrayLevelEmphasis,  original_glszm_ZoneEntropy,  original_glszm_ZonePercentage,  original_glszm_ZoneVariance | 16 |
| GLDM | original_gldm_DependenceEntropy,  original_gldm_DependenceNonUniformity,  original_gldm_DependenceNonUniformityNormalized,  original_gldm_DependenceVariance,  original_gldm_GrayLevelNonUniformity,  original_gldm_GrayLevelVariance,  original_gldm_HighGrayLevelEmphasis,  original_gldm_LargeDependenceEmphasis,  original_gldm_LargeDependenceHighGrayLevelEmphasis,  original_gldm_LargeDependenceLowGrayLevelEmphasis,  original_gldm_LowGrayLevelEmphasis,  original_gldm_SmallDependenceEmphasis,  original_gldm_SmallDependenceHighGrayLevelEmphasis,  original_gldm_SmallDependenceLowGrayLevelEmphasis | 14 |

Note. GLCM, grey level co-occurrence matrix. GLRLM, grey-level run length matrix. GLSZM, grey-level size zone matrix. GLDM, gray level dependence matrix.
